# Synthesis and crystal structures of [Al(H_2_O)_6_](SO_4_)NO_3_·2H_2_O and [Al(H_2_O)_6_](SO_4_)Cl·H_2_O

**DOI:** 10.1107/S2056989020015741

**Published:** 2021-01-01

**Authors:** Fredric G. Svensson

**Affiliations:** a Department of Molecular Sciences, Swedish University of Agricultural Sciences, Box 7015, 750 07 Uppsala, Sweden

**Keywords:** crystal structure, aluminium, double salt, hydrogen bonding

## Abstract

Two novel aluminium double salts, [Al(H_2_O)_6_](SO_4_)NO_3_·2H_2_O, hexa­qua­alumin­ium sulfate nitrate dihydrate, (**1**), and [Al(H_2_O)_6_](SO_4_)Cl·H_2_O, hexaqua­aluminium sulfate chloride hydrate, (**2**), were obtained in the form of single crystals. Their crystal structures are each based on an octa­hedral [Al(H_2_O)_6_]^3+^ unit and both structures have in common one charge-balancing SO_4_^2−^ anion. The final positive charge from the aluminium(III) cation is balanced by an NO_3_^−^ or a Cl^−^ anion for (**1**) and (**2**), respectively. Compound (**1**) further contains two unligated water mol­ecules while compound (**2**) only contains one unligated water mol­ecule. In the crystal structures, all components are spatially separated and inter­actions are mediated *via* medium–strong hydrogen bonding, compared to many other reported aluminium sulfates where corner-sharing of the building units is common. The two compounds represent rare cases where one aluminium(III) cation is charge-balanced by two different anions.

## Abstract 2

Common features in the crystal structures of [Al(H_2_O)_6_](SO_4_)NO_3_·2H_2_O and [Al(H_2_O)_6_](SO_4_)Cl·H_2_O are [Al(H_2_O)_6_]^3+^ octa­hedra and sulfate tetra­hedra. These components, the remaining anion (NO_3_^−^ and Cl^−^, respectively) and lattice water mol­ecules are separated from each other. Inter­actions are mediated *via* medium–strong hydrogen bonding.

## Chemical context   

Aluminium is one of the most common elements in Earth’s crust and is predominantly found in oxides and silicates. The far most common oxidation state for inorganic compounds is +III. Aluminium is found in many double salts with numerous other cations and sulfate, such as the industrially important alums *M*Al(SO_4_)_2_·12H_2_O (*M* = monovalent cation; Greenwood & Earnshaw, 1997 ▸). At low pH, aluminium mainly exists in solution as the [Al(H_2_O)_6_]^3+^ cation (Hay & Myneni, 2008 ▸).

One of the title compounds, [Al(H_2_O)_6_](SO_4_)NO_3_·2H_2_O, (**1**), was obtained as an unintentional side product when attempting to synthesize an aluminium-modified bis­muth-titanium oxo-complex. Efforts to obtain (**1**) by other routes resulted in the formation of [Al(H_2_O)_6_]SO_4_Cl·H_2_O (**2**).

## Structural commentary   

**Figure 1:**
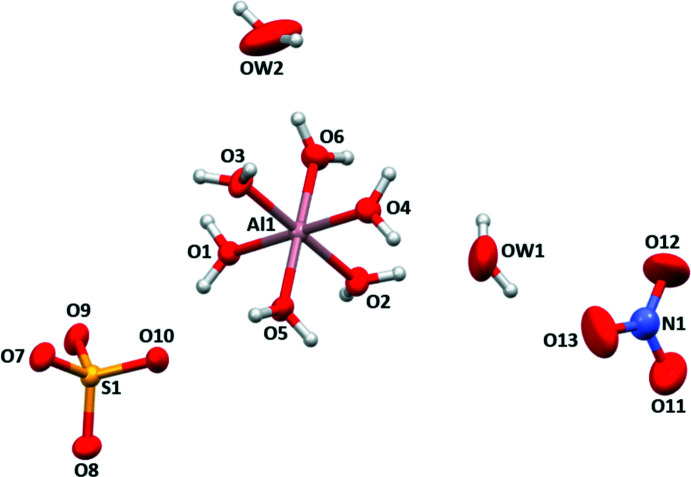
The asymmetric unit of (**1**), representing the building units. Displacement ellipsoids are drawn at the 50% probability level.

**Figure 2:**
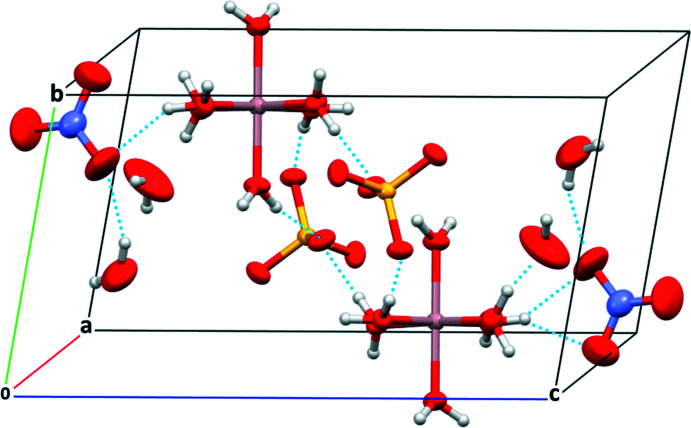
Packing in the crystal structure of compound (**1**). Hydrogen bonding is indicated by dotted lines.

The crystal structure of (**1**) comprises an [Al(H_2_O)_6_]^3+^ cation charge-balanced by one sulfate and one nitrate anion as well as two unligated water mol­ecules; all building units are separated from each other (Fig. 1 ▸). Bond lengths in the components are summarized in Table 1 ▸. The aqua ligands (O1–O6) of the complex cations serve as hydrogen-bonding donor groups. They connect through O—H⋯O hydrogen bonds to the two types of anions and to the two unbound water mol­ecules, forming a three-dimensional network (Fig. 2 ▸, Table 2 ▸). Hydrogen bonds involving H8 and H12 are bifurcated. The water mol­ecules O*W*1 and O*W*2 likewise serve as donor groups, whereby O*W*1 hydrogen-bonds to the nitrate anion (O12, O13) and to the second water mol­ecule O*W*2. The latter hydrogen bond involving H14 is also bifurcated. Inter­estingly, O*W*2 shows only one hydrogen bond to a nitrate anion (H16⋯O12); the second H atom (H15) is not engaged in hydrogen-bonding. The H⋯O distances involving the [Al(H_2_O)_6_]^3+^ group are between 1.76 (3) and 2.35 (3) Å and thus can be considered as medium–strong whereas the H⋯O distances [2.05 (2) to 2.55 (3) Å] involving the unbound water mol­ecules as donor groups indicate much weaker hydrogen bonds.

**Figure 3:**
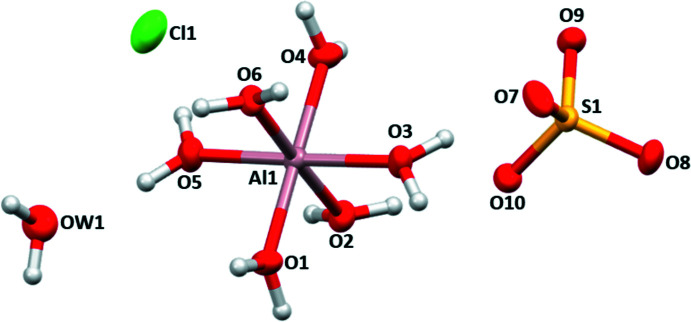
The asymmetric unit of (**2**), representing the building units. Displacement ellipsoids are drawn at the 50% probability level.

**Figure 4:**
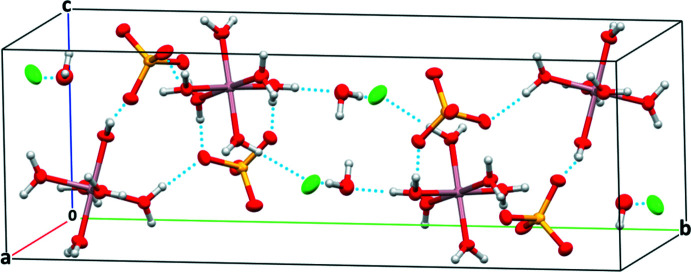
Packing in the crystal structure of compound (**2**). Hydrogen bonding is indicated by dotted lines.

In the crystal structure of compound (**2**), the charge-balancing nitrate anion of (**1**) is exchanged for a chloride anion, and the formula unit only contains one additional water mol­ecule (Fig. 3 ▸). Table 3 ▸ collates bond lengths of the individual building units. The [Al(H_2_O)_6_]^3+^ cation donates hydrogen bonds through the aqua ligands (O1– O6) to the sulfate group, the unligated water mol­ecule and to the chloride anion, resulting in a three-dimensional network (Fig. 4 ▸, Table 4 ▸). Each sulfate group is hydrogen-bonded to four different [Al(H_2_O)_6_]^3+^ cations, and the unbound water mol­ecule exclusively hydrogen-bonds to the chloride anions, partly with a bifurcated bond. The O⋯H distances vary between 1.726 (11) and 1.917 (11) Å and thus are slightly stronger than in (**1**).

According to the Pearson concept, sulfate, nitrate, and chloride are all considered inter­mediate hard bases while Al^3+^ is a hard acid. The higher charge (2+) of the sulfate group compared to the nitrate group and chloride is a likely reason that the sulfate group is present in both structures while the two latter ones can be inter­changed, possibly related to their relative abundance. The chloride ions in the reaction mixture of (**1**) might also have been bonded to the titanium(IV) and bis­muth(III) cations, preventing the formation of (**2**). In particular Bi^3+^ tends to form insoluble BiOCl. Furthermore, (**1**) contains two extra water mol­ecules while (**2**) only contains one of them. The average Al—O bond lengths are 1.880 and 1.884 Å for (**1**) and (**2**), respectively, which is slightly shorter than the literature average distance of 1.90 Å (Hay & Myneni, 2008 ▸; Veillard, 1977 ▸).

Structures of aluminium sulfate, Al_2_(SO_4_)_3_, and derivatives thereof have been reported with different amounts of additional structural water and varying connectivities. Sabelli & Ferroni (1978 ▸) reported an aluminium sulfate structure (Al_2_(OH)_4_SO_4_·7H_2_O) where six hydrated aluminum(III) ions are connected *via* edge- and face sharing. These aluminum ‘hexa­mers’ are linked *via* hydrogen bonding with unligated water and sulfate ions. In the crystal structure of Al_2_(SO_4_)_3_·8H_2_O, hydrated aluminum(III) ions are connected *via* corner sharing with sulfate groups and a rather extensive hydrogen-bond network between sulfate, aqua ligands, and unligated, structural water mol­ecules (Fischer *et al.*, 1996 ▸). In the Al(SO_4_)OH structure reported by Anderson *et al.* (2015 ▸), each sulfate group connects three different aluminium(III) ions *via* corner sharing. The structures of the two reported compounds herein are more open and the principal building units are only connected *via* hydrogen bonding, which may be due to the presence of another anion (NO_3_^−^/Cl^−^).

## Database survey   

According to a database survey using the Inorganic Crystal Structure Database (ICSD), aluminium compounds with an additional cation charge-balanced by sulfate anions appear to be common [*e.g*. KAl(SO_4_)_2_, FeAl(SO_4_)_3_ (Demartin *et al.*, 2010 ▸), or CsAl(SO_4_)_2_ (Beattie *et al.*, 1981 ▸)]. However, compounds with aluminium as the single cation but with two different anions were found to be much less common although examples include Al(H_2_PO_4_)_2_F (Parnham & Morris, 2006 ▸) or Al(SO_4_)OH (Anderson *et al.*, 2015 ▸).

## Synthesis and crystallization   

Compound (**1**) was obtained by mixing equimolar solutions of TiOSO_4_ (Aldrich) and Bi(NO_3_)_3_·5H_2_O (Aldrich), both dissolved in 1 *M* nitric acid (Sigma–Aldrich), and two equivalents of AlCl_3_·6H_2_O (Mallinckrodt Chemical Works) dissolved in 1 *M* hydro­chloric acid (Sigma–Aldrich). Colorless needle-shaped crystals formed on a glass substrate after about a week of slow evaporation of the solvent at room temperature. Elemental analysis by energy-dispersive X-ray spectroscopy using a Hitachi TM-1000 scanning electron microscope with an Oxford Instruments EDS system revealed a molar Al:S ratio of 1.37 (expected 1:1). In an attempt to synthesize compound (**1**) by a direct route, aluminium(III) chloride was changed to aluminium(III) lactate to avoid chloride ions. This resulted in formation of crystals with very poor quality that were not suitable for X-ray diffraction.

Compound (**2**) was obtained by dissolving 1 *M* AlCl_3_·6H_2_O in 1 ml of 1 *M* hydro­chloric acid and adding one equivalent of 1 *M* sulfuric acid (Sigma–Aldrich), or making a 1 *M* AlCl_3_·6H_2_O solution in 0.5 ml of 1 *M* H_2_SO_2_ plus 0.5 ml of 1 *M* HNO_3_. The solution was poured into a Petri dish and left for slow evaporation. After a few days of evaporation of the solvent, colorless block-shaped crystals suitable for single X-ray crystal diffraction were obtained. The crystals were somewhat fragile. EDS analysis of (**2**) revealed an S:Al:Cl molar composition of 0.9:0.9:1.17 (expected 1:1:1).

For the data collection, both types of crystals were mounted on a glass needle and protected by a layer of paraffin oil.

## Refinement   

Crystal data, data collection and structure refinement details are summarized in Table 5 ▸. In each of the two structures, all hydrogen atoms were discernible in difference-Fourier maps. They were refined with O—H distance restraints of 0.85 (1) Å and a common *U*_iso_(H) parameter. Reasonable geometries for the unligated water water mol­ecules were ensured by using restrained H⋯H distances of 1.55 (1) Å.

## Supplementary Material

Crystal structure: contains datablock(s) global, 1, 2. DOI: 10.1107/S2056989020015741/wm5585sup1.cif

CCDC references: 2030818, 2028868

Additional supporting information:  crystallographic information; 3D view; checkCIF report

## Figures and Tables

**Table 1:** Selected bond lengths (Å) for (**1**)

Al1—O6	1.869 (2)	S1—O10	1.466 (2)
Al1—O5	1.872 (2)	S1—O7	1.470 (2)
Al1—O2	1.876 (2)	S1—O9	1.479 (2)
Al1—O3	1.880 (2)	N1—O12	1.209 (4)
Al1—O1	1.880 (2)	N1—O11	1.225 (4)
Al1—O4	1.887 (2)	N1—O13	1.232 (4)
S1—O8	1.464 (2)		

**Table 2:** Hydrogen-bond geometry (Å, °) for (**1**)

*D*—H⋯*A*	*D*—H	H⋯*A*	*D*⋯*A*	*D*—H⋯*A*
O1—H1⋯O10	0.85 (1)	1.78 (1)	2.627 (3)	173 (4)
O1—H2⋯O7^i^	0.85 (1)	1.85 (1)	2.689 (3)	171 (4)
O2—H3⋯O8^ii^	0.85 (1)	1.84 (1)	2.684 (3)	176 (4)
O2—H4⋯O*W*1	0.85 (1)	1.76 (1)	2.600 (3)	171 (4)
O3—H5⋯O7^iii^	0.85 (1)	1.83 (1)	2.675 (3)	178 (4)
O3—H6⋯O9^i^	0.85 (1)	1.83 (1)	2.670 (3)	169 (4)
O4—H7⋯O8^iv^	0.85 (1)	1.91 (1)	2.745 (3)	168 (4)
O4—H8⋯O11^v^	0.85 (1)	2.12 (2)	2.884 (4)	150 (4)
O4—H8⋯O13^v^	0.85 (1)	2.13 (3)	2.870 (4)	147 (4)
O5—H9⋯O9^vi^	0.85 (1)	1.80 (1)	2.650 (3)	179 (4)
O5—H10⋯O10^iv^	0.85 (1)	1.79 (1)	2.640 (3)	175 (4)
O6—H11⋯O*W*2	0.85 (1)	1.79 (2)	2.596 (4)	160 (4)
O6—H12⋯O11^vii^	0.85 (1)	2.35 (3)	3.044 (4)	139 (3)
O6—H12⋯O12^vii^	0.85 (1)	2.06 (2)	2.876 (4)	162 (4)
O*W*1—H13⋯O13	0.86 (1)	2.05 (2)	2.850 (5)	155 (3)
O*W*1—H14⋯O12^vii^	0.86 (1)	2.52 (4)	3.109 (5)	127 (4)
O*W*1—H14⋯O*W*2^viii^	0.86 (1)	2.55 (4)	3.073 (6)	120 (3)
O*W*2—H16⋯O12^viii^	0.86 (1)	2.20 (3)	2.908 (5)	139 (3)

Symmetry codes: (i) 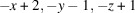; (ii) 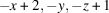; (iii) 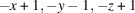; (iv) 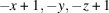; (v) 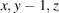; (vi) 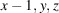; (vii) 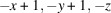; (viii) 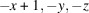.

**Table 3:** Selected bond lengths (Å) for (**2**)

Al1—O3	1.8624 (17)	Al1—O2	1.8940 (17)
Al1—O5	1.8718 (18)	S1—O10	1.4670 (16)
Al1—O6	1.8752 (17)	S1—O9	1.4672 (16)
Al1—O1	1.8798 (17)	S1—O8	1.4753 (16)
Al1—O4	1.8855 (17)	S1—O7	1.4767 (16)

**Table 4:** Hydrogen-bond geometry (Å, °) for (**2**)

*D*—H⋯*A*	*D*—H	H⋯*A*	*D*⋯*A*	*D*—H⋯*A*
O1—H1⋯O9^i^	0.85 (1)	1.88 (1)	2.714 (2)	165 (3)
O1—H2⋯O8^ii^	0.85 (1)	1.85 (1)	2.690 (2)	170 (3)
O2—H3⋯O*W*1^iii^	0.85 (1)	1.85 (1)	2.692 (2)	178 (3)
O2—H4⋯O10	0.85 (1)	1.92 (1)	2.767 (2)	177 (3)
O3—H5⋯O7^iv^	0.85 (1)	1.78 (1)	2.629 (2)	174 (3)
O3—H6⋯O7	0.85 (1)	1.73 (1)	2.578 (2)	176 (3)
O4—H7⋯Cl1^v^	0.85 (1)	2.18 (1)	3.0311 (18)	177 (3)
O4—H8⋯O10^vi^	0.85 (1)	1.83 (1)	2.669 (2)	176 (3)
O5—H9⋯O*W*1	0.85 (1)	1.82 (1)	2.650 (2)	166 (3)
O5—H10⋯Cl1	0.85 (1)	2.17 (1)	3.0120 (18)	171 (3)
O6—H11⋯O8^vii^	0.85 (1)	1.83 (1)	2.672 (2)	172 (3)
O6—H12⋯O9^ii^	0.85 (1)	1.83 (1)	2.671 (2)	171 (3)
O*W*1—H13⋯Cl1^viii^	0.85 (1)	2.33 (2)	3.083 (2)	149 (3)
O*W*1—H14⋯Cl1^i^	0.84 (1)	2.68 (2)	3.390 (2)	143 (3)
O*W*1—H14⋯Cl1^iii^	0.84 (1)	2.74 (3)	3.280 (2)	123 (3)

Symmetry codes: (i) 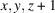; (ii) 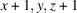; (iii) 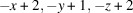; (iv) 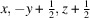; (v) 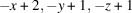; (vi) 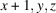; (vii) 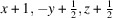; (viii) 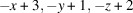.

**Table 5:** Experimental details

	(**1**)	(**2**)
Crystal data		
Chemical formula	[Al(H_2_O)_6_](NO_3_)(SO_4_)·2H_2_O	[Al(H_2_O)_6_]Cl(SO_4_)·H_2_O
*M*_r_	329.18	284.60
Crystal system, space group	Triclinic, *P* 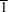	Monoclinic, *P*2_1_/*c*
Temperature (K)	296	296
*a*, *b*, *c* (Å)	6.088 (4), 7.377 (5), 13.721 (9)	6.1640 (14), 22.933 (5), 7.2876 (14)
α, β, γ (°)	77.340 (6), 89.561 (7), 82.712 (7)	90, 97.328 (2), 90
*V* (Å^3^)	596.3 (7)	1021.8 (4)
*Z*	2	4
Radiation type	Mo *K*α	Mo *K*α
μ (mm^−1^)	0.43	0.71
Crystal size (mm)	0.20 × 0.02 × 0.02	0.20 × 0.10 × 0.10

Data collection		
Diffractometer	Bruker APEXII CCD	Bruker APEXII CCD
Absorption correction	Multi-scan (*SADABS*; Bruker, 2015 ▸)	Multi-scan (*SADABS*; Bruker, 2015 ▸)
*T*_min_, *T*_max_	0.919, 0.992	0.872, 0.933
No. of measured, independent and observed [*I* > 2σ(*I*)] reflections	4506, 1642, 1519	8454, 1457, 1304
*R*_int_	0.026	0.044
θ_max_ (°)	23.4	23.3
(sin θ/λ)_max_ (Å^−1^)	0.559	0.556

Refinement		
*R*[*F* ^2^ > 2σ(*F* ^2^)], *wR*(*F* ^2^), *S*	0.034, 0.090, 1.10	0.024, 0.064, 1.03
No. of reflections	1642	1457
No. of parameters	213	170
No. of restraints	97	14
H-atom treatment	H atoms treated by a mixture of independent and constrained refinement	H atoms treated by a mixture of independent and constrained refinement
Δρ_max_, Δρ_min_ (e Å^−3^)	0.50, −0.38	0.20, −0.29

Computer programs: *APEX2* and *SAINT* (Bruker, 2015 ▸), *SHELXS* (Sheldrick, 2008 ▸), *SHELXL* (Sheldrick, 2015 ▸), *Mercury* (Macrae *et al.*, 2020 ▸) and *publCIF* (Westrip, 2010 ▸).

## supplementary crystallographic information

### Hexaquaaluminium sulfate nitrate dihydrate (1) Crystal data

**Table:**

[Al(H_2_O)_6_](NO_3_)(SO_4_)·2H_2_O	*Z* = 2
*M**_r_* = 329.18	*F*(000) = 344
triclinic, *P*1	*D*_x_ = 1.833 Mg m^−^^3^
*a* = 6.088 (4) Å	Mo *K*α radiation, λ = 0.71073 Å
*b* = 7.377 (5) Å	Cell parameters from 3914 reflections
*c* = 13.721 (9) Å	θ = 2.9–23.4°
α = 77.340 (6)°	µ = 0.43 mm^−^^1^
β = 89.561 (7)°	*T* = 296 K
γ = 82.712 (7)°	Needle, colorless
*V* = 596.3 (7) Å^3^	0.20 × 0.02 × 0.02 mm

### Hexaquaaluminium sulfate nitrate dihydrate (1) Data collection

**Table:**

Bruker APEXII CCD diffractometer	1642 independent reflections
Radiation source: fine-focus sealed tube	1519 reflections with *I* > 2σ(*I*)
Graphite monochromator	*R*_int_ = 0.026
φ and ω scans	θ_max_ = 23.4°, θ_min_ = 2.9°
Absorption correction: multi-scan (*SADABS*; Bruker, 2015)	*h* = −6→6
*T*_min_ = 0.919, *T*_max_ = 0.992	*k* = −8→8
4506 measured reflections	*l* = −15→15

### Hexaquaaluminium sulfate nitrate dihydrate (1) Refinement

**Table:**

Refinement on *F*^2^	Secondary atom site location: difference Fourier map
Least-squares matrix: full	Hydrogen site location: inferred from neighbouring sites
*R*[*F*^2^ > 2σ(*F*^2^)] = 0.034	H atoms treated by a mixture of independent and constrained refinement
*wR*(*F*^2^) = 0.090	*w* = 1/[σ^2^(*F*_o_^2^) + (0.0404*P*)^2^ + 0.6205*P*] where *P* = (*F*_o_^2^ + 2*F*_c_^2^)/3
*S* = 1.10	(Δ/σ)_max_ < 0.001
1642 reflections	Δρ_max_ = 0.50 e Å^−^^3^
213 parameters	Δρ_min_ = −0.38 e Å^−^^3^
97 restraints	Extinction correction: SHELXL, Fc^*^=kFc[1+0.001xFc^2^λ^3^/sin(2θ)]^-1/4^
Primary atom site location: structure-invariant direct methods	Extinction coefficient: 0.021 (4)

### Hexaquaaluminium sulfate nitrate dihydrate (1) Fractional atomic coordinates and isotropic or equivalent isotropic displacement parameters (Å^2^)

**Table:**

	*x*	*y*	*z*	*U*_iso_*/*U*_eq_	
Al1	0.49771 (11)	−0.14486 (9)	0.30419 (5)	0.0196 (2)	
S1	1.01766 (9)	−0.31760 (8)	0.62701 (4)	0.0206 (2)	
O1	0.7790 (3)	−0.2120 (2)	0.36672 (13)	0.0244 (4)	
O2	0.5436 (3)	0.1080 (2)	0.27387 (14)	0.0283 (4)	
O3	0.4467 (3)	−0.3969 (2)	0.33515 (14)	0.0290 (5)	
O4	0.2144 (3)	−0.0792 (3)	0.24229 (14)	0.0286 (4)	
O5	0.3748 (3)	−0.1058 (2)	0.42456 (13)	0.0270 (4)	
O6	0.6292 (3)	−0.1776 (3)	0.18465 (14)	0.0316 (5)	
O7	0.9553 (3)	−0.4996 (2)	0.67900 (14)	0.0288 (4)	
O8	1.0542 (3)	−0.2042 (3)	0.69915 (14)	0.0315 (5)	
O9	1.2243 (3)	−0.3510 (2)	0.57270 (14)	0.0322 (5)	
O10	0.8384 (3)	−0.2193 (3)	0.55706 (14)	0.0353 (5)	
N1	0.2189 (5)	0.8543 (4)	0.0100 (2)	0.0446 (7)	
O11	0.2452 (5)	1.0059 (4)	0.0276 (2)	0.0725 (8)	
O12	0.2331 (6)	0.8332 (5)	−0.0748 (2)	0.0901 (10)	
O13	0.1767 (6)	0.7326 (4)	0.0821 (2)	0.0823 (9)	
OW1	0.3034 (5)	0.3374 (4)	0.1321 (2)	0.0754 (8)	
OW2	0.7579 (7)	−0.4488 (6)	0.0962 (4)	0.1134 (14)	
H1	0.809 (7)	−0.220 (6)	0.4280 (11)	0.069 (3)*	
H2	0.867 (6)	−0.294 (4)	0.347 (3)	0.069 (3)*	
H3	0.672 (3)	0.139 (6)	0.279 (3)	0.069 (3)*	
H4	0.478 (6)	0.187 (4)	0.225 (2)	0.069 (3)*	
H5	0.320 (3)	−0.432 (6)	0.332 (3)	0.069 (3)*	
H6	0.540 (5)	−0.487 (4)	0.364 (3)	0.069 (3)*	
H7	0.117 (5)	0.004 (4)	0.256 (3)	0.069 (3)*	
H8	0.193 (7)	−0.087 (6)	0.1826 (13)	0.069 (3)*	
H9	0.327 (7)	−0.183 (5)	0.473 (2)	0.069 (3)*	
H10	0.313 (6)	0.001 (3)	0.431 (3)	0.069 (3)*	
H11	0.648 (7)	−0.279 (3)	0.164 (3)	0.069 (3)*	
H12	0.657 (7)	−0.083 (4)	0.141 (2)	0.069 (3)*	
H13	0.265 (7)	0.451 (2)	0.137 (3)	0.069 (3)*	
H14	0.367 (7)	0.298 (4)	0.083 (2)	0.069 (3)*	
H15	0.616 (2)	−0.421 (5)	0.091 (3)	0.069 (3)*	
H16	0.831 (5)	−0.555 (3)	0.092 (3)	0.069 (3)*	

### Hexaquaaluminium sulfate nitrate dihydrate (1) Atomic displacement parameters (Å^2^)

**Table:**

	*U*^11^	*U*^22^	*U*^33^	*U*^12^	*U*^13^	*U*^23^
Al1	0.0169 (4)	0.0196 (4)	0.0222 (4)	−0.0027 (3)	−0.0003 (3)	−0.0041 (3)
S1	0.0178 (4)	0.0187 (4)	0.0248 (4)	−0.0008 (2)	−0.0005 (2)	−0.0046 (2)
O1	0.0199 (9)	0.0249 (9)	0.0279 (10)	0.0008 (7)	−0.0036 (7)	−0.0068 (8)
O2	0.0250 (10)	0.0236 (10)	0.0349 (11)	−0.0063 (8)	−0.0027 (8)	−0.0011 (8)
O3	0.0202 (9)	0.0205 (10)	0.0453 (12)	−0.0043 (7)	−0.0014 (8)	−0.0040 (8)
O4	0.0209 (9)	0.0333 (11)	0.0323 (10)	0.0002 (8)	−0.0040 (8)	−0.0106 (8)
O5	0.0313 (10)	0.0220 (10)	0.0255 (10)	0.0014 (8)	0.0054 (8)	−0.0032 (7)
O6	0.0321 (10)	0.0358 (11)	0.0268 (10)	−0.0035 (9)	0.0052 (8)	−0.0075 (8)
O7	0.0229 (9)	0.0220 (9)	0.0406 (11)	−0.0050 (7)	0.0033 (8)	−0.0039 (8)
O8	0.0263 (9)	0.0310 (10)	0.0411 (11)	−0.0042 (8)	−0.0033 (8)	−0.0158 (8)
O9	0.0282 (10)	0.0257 (10)	0.0373 (11)	0.0021 (8)	0.0103 (8)	0.0012 (8)
O10	0.0368 (11)	0.0348 (11)	0.0313 (10)	0.0134 (8)	−0.0101 (8)	−0.0107 (8)
N1	0.0476 (15)	0.0430 (16)	0.0396 (16)	0.0011 (12)	0.0073 (12)	−0.0053 (12)
O11	0.0610 (16)	0.0542 (16)	0.103 (2)	−0.0068 (13)	−0.0250 (15)	−0.0186 (15)
O12	0.126 (3)	0.100 (2)	0.0455 (16)	0.008 (2)	0.0206 (16)	−0.0325 (16)
O13	0.098 (2)	0.0772 (19)	0.0586 (17)	−0.0316 (17)	−0.0027 (15)	0.0257 (15)
OW1	0.085 (2)	0.0536 (16)	0.0713 (19)	0.0160 (15)	−0.0088 (16)	0.0071 (14)
OW2	0.092 (3)	0.107 (3)	0.171 (4)	−0.005 (2)	0.016 (3)	−0.101 (3)

### Hexaquaaluminium sulfate nitrate dihydrate (1) Geometric parameters (Å, º)

**Table:**

Al1—O6	1.869 (2)	S1—O10	1.466 (2)
Al1—O5	1.872 (2)	S1—O7	1.470 (2)
Al1—O2	1.876 (2)	S1—O9	1.479 (2)
Al1—O3	1.880 (2)	N1—O12	1.209 (4)
Al1—O1	1.880 (2)	N1—O11	1.225 (4)
Al1—O4	1.887 (2)	N1—O13	1.232 (4)
S1—O8	1.464 (2)		
			
O6—Al1—O5	177.66 (9)	O2—Al1—O4	90.05 (8)
O6—Al1—O2	90.30 (9)	O3—Al1—O4	89.21 (8)
O5—Al1—O2	87.90 (8)	O1—Al1—O4	179.48 (9)
O6—Al1—O3	90.38 (9)	O8—S1—O10	109.31 (11)
O5—Al1—O3	91.45 (9)	O8—S1—O7	110.17 (12)
O2—Al1—O3	179.02 (8)	O10—S1—O7	108.97 (12)
O6—Al1—O1	88.42 (9)	O8—S1—O9	109.41 (12)
O5—Al1—O1	90.10 (9)	O10—S1—O9	110.42 (12)
O2—Al1—O1	90.37 (8)	O7—S1—O9	108.55 (11)
O3—Al1—O1	90.36 (8)	O12—N1—O11	119.5 (3)
O6—Al1—O4	91.88 (9)	O12—N1—O13	124.4 (3)
O5—Al1—O4	89.61 (9)	O11—N1—O13	116.2 (3)

### Hexaquaaluminium sulfate nitrate dihydrate (1) Hydrogen-bond geometry (Å, º)

**Table:**

*D*—H···*A*	*D*—H	H···*A*	*D*···*A*	*D*—H···*A*
O1—H1···O10	0.85 (1)	1.78 (1)	2.627 (3)	173 (4)
O1—H2···O7^i^	0.85 (1)	1.85 (1)	2.689 (3)	171 (4)
O2—H3···O8^ii^	0.85 (1)	1.84 (1)	2.684 (3)	176 (4)
O2—H4···O*W*1	0.85 (1)	1.76 (1)	2.600 (3)	171 (4)
O3—H5···O7^iii^	0.85 (1)	1.83 (1)	2.675 (3)	178 (4)
O3—H6···O9^i^	0.85 (1)	1.83 (1)	2.670 (3)	169 (4)
O4—H7···O8^iv^	0.85 (1)	1.91 (1)	2.745 (3)	168 (4)
O4—H8···O11^v^	0.85 (1)	2.12 (2)	2.884 (4)	150 (4)
O4—H8···O13^v^	0.85 (1)	2.13 (3)	2.870 (4)	147 (4)
O5—H9···O9^vi^	0.85 (1)	1.80 (1)	2.650 (3)	179 (4)
O5—H10···O10^iv^	0.85 (1)	1.79 (1)	2.640 (3)	175 (4)
O6—H11···O*W*2	0.85 (1)	1.79 (2)	2.596 (4)	160 (4)
O6—H12···O11^vii^	0.85 (1)	2.35 (3)	3.044 (4)	139 (3)
O6—H12···O12^vii^	0.85 (1)	2.06 (2)	2.876 (4)	162 (4)
O*W*1—H13···O13	0.86 (1)	2.05 (2)	2.850 (5)	155 (3)
O*W*1—H14···O12^vii^	0.86 (1)	2.52 (4)	3.109 (5)	127 (4)
O*W*1—H14···O*W*2^viii^	0.86 (1)	2.55 (4)	3.073 (6)	120 (3)
O*W*2—H16···O12^viii^	0.86 (1)	2.20 (3)	2.908 (5)	139 (3)

Symmetry codes: (i) −*x*+2, −*y*−1, −*z*+1; (ii) −*x*+2, −*y*, −*z*+1; (iii) −*x*+1, −*y*−1, −*z*+1; (iv) −*x*+1, −*y*, −*z*+1; (v) *x*, *y*−1, *z*; (vi) *x*−1, *y*, *z*; (vii) −*x*+1, −*y*+1, −*z*; (viii) −*x*+1, −*y*, −*z*.

### Hexaquaaluminium sulfate chloride monohydrate (2) Crystal data

**Table:**

[Al(H_2_O)_6_]Cl(SO_4_)·H_2_O	*F*(000) = 592
*M**_r_* = 284.60	*D*_x_ = 1.850 Mg m^−^^3^
monoclinic, *P*2_1_/*c*	Mo *K*α radiation, λ = 0.71073 Å
*a* = 6.1640 (14) Å	Cell parameters from 3645 reflections
*b* = 22.933 (5) Å	θ = 3.0–23.3°
*c* = 7.2876 (14) Å	µ = 0.71 mm^−^^1^
β = 97.328 (2)°	*T* = 296 K
*V* = 1021.8 (4) Å^3^	Block, coloress
*Z* = 4	0.20 × 0.10 × 0.10 mm

### Hexaquaaluminium sulfate chloride monohydrate (2) Data collection

**Table:**

Bruker APEXII CCD diffractometer	1457 independent reflections
Radiation source: fine-focus sealed tube	1304 reflections with *I* > 2σ(*I*)
Graphite monochromator	*R*_int_ = 0.044
φ and ω scans	θ_max_ = 23.3°, θ_min_ = 3.0°
Absorption correction: multi-scan (*SADABS*; Bruker, 2015)	*h* = −6→6
*T*_min_ = 0.872, *T*_max_ = 0.933	*k* = −25→25
8454 measured reflections	*l* = −8→8

### Hexaquaaluminium sulfate chloride monohydrate (2) Refinement

**Table:**

Refinement on *F*^2^	Primary atom site location: structure-invariant direct methods
Least-squares matrix: full	Secondary atom site location: difference Fourier map
*R*[*F*^2^ > 2σ(*F*^2^)] = 0.024	Hydrogen site location: inferred from neighbouring sites
*wR*(*F*^2^) = 0.064	H atoms treated by a mixture of independent and constrained refinement
*S* = 1.03	*w* = 1/[σ^2^(*F*_o_^2^) + (0.0256*P*)^2^ + 0.8819*P*] where *P* = (*F*_o_^2^ + 2*F*_c_^2^)/3
1457 reflections	(Δ/σ)_max_ < 0.001
170 parameters	Δρ_max_ = 0.20 e Å^−^^3^
14 restraints	Δρ_min_ = −0.29 e Å^−^^3^

### Hexaquaaluminium sulfate chloride monohydrate (2) Fractional atomic coordinates and isotropic or equivalent isotropic displacement parameters (Å^2^)

**Table:**

	*x*	*y*	*z*	*U*_iso_*/*U*_eq_	
Al1	0.94442 (10)	0.36368 (3)	0.83030 (9)	0.01735 (19)	
S1	0.41721 (9)	0.32296 (2)	0.34162 (7)	0.01806 (17)	
O1	0.8883 (3)	0.34621 (7)	1.0718 (2)	0.0242 (4)	
O2	0.6779 (3)	0.40544 (7)	0.7845 (2)	0.0236 (4)	
O3	0.7994 (3)	0.29620 (7)	0.7410 (2)	0.0236 (4)	
O4	1.0001 (3)	0.38033 (7)	0.5873 (2)	0.0243 (4)	
O5	1.0925 (3)	0.43174 (7)	0.9146 (2)	0.0262 (4)	
O6	1.2075 (3)	0.32177 (7)	0.8685 (2)	0.0218 (4)	
O7	0.5853 (3)	0.28049 (7)	0.4172 (2)	0.0288 (4)	
O8	0.2061 (2)	0.29253 (7)	0.2976 (2)	0.0251 (4)	
O9	0.4791 (3)	0.34900 (7)	0.1719 (2)	0.0257 (4)	
O10	0.3963 (2)	0.36795 (7)	0.4814 (2)	0.0237 (4)	
Cl1	1.22318 (11)	0.52579 (3)	0.66184 (10)	0.0399 (2)	
OW1	1.2769 (3)	0.47795 (8)	1.2298 (3)	0.0329 (4)	
H1	0.768 (3)	0.3524 (14)	1.115 (4)	0.054 (3)*	
H2	0.977 (4)	0.3286 (12)	1.152 (3)	0.054 (3)*	
H3	0.691 (5)	0.4422 (5)	0.783 (5)	0.054 (3)*	
H4	0.588 (4)	0.3942 (14)	0.693 (3)	0.054 (3)*	
H5	0.734 (5)	0.2727 (11)	0.805 (4)	0.054 (3)*	
H6	0.734 (5)	0.2915 (14)	0.632 (2)	0.054 (3)*	
H7	0.933 (4)	0.4067 (10)	0.520 (4)	0.054 (3)*	
H8	1.126 (3)	0.3750 (14)	0.557 (4)	0.054 (3)*	
H9	1.143 (5)	0.4415 (13)	1.025 (2)	0.054 (3)*	
H10	1.130 (5)	0.4554 (11)	0.834 (3)	0.054 (3)*	
H11	1.215 (5)	0.2853 (5)	0.855 (4)	0.054 (3)*	
H12	1.304 (4)	0.3305 (14)	0.957 (3)	0.054 (3)*	
H13	1.400 (3)	0.4634 (13)	1.270 (4)	0.054 (3)*	
H14	1.220 (5)	0.4762 (14)	1.329 (3)	0.054 (3)*	

### Hexaquaaluminium sulfate chloride monohydrate (2) Atomic displacement parameters (Å^2^)

**Table:**

	*U*^11^	*U*^22^	*U*^33^	*U*^12^	*U*^13^	*U*^23^
Al1	0.0135 (4)	0.0205 (4)	0.0175 (4)	−0.0001 (3)	0.0002 (3)	0.0011 (3)
S1	0.0136 (3)	0.0220 (3)	0.0178 (3)	0.0011 (2)	−0.0007 (2)	−0.0008 (2)
O1	0.0173 (9)	0.0370 (10)	0.0184 (9)	0.0042 (8)	0.0026 (7)	0.0054 (7)
O2	0.0171 (9)	0.0264 (9)	0.0260 (9)	0.0017 (7)	−0.0017 (7)	0.0001 (8)
O3	0.0230 (9)	0.0267 (10)	0.0194 (9)	−0.0064 (7)	−0.0034 (7)	0.0031 (7)
O4	0.0193 (9)	0.0306 (10)	0.0236 (9)	0.0051 (7)	0.0052 (7)	0.0074 (7)
O5	0.0265 (9)	0.0250 (10)	0.0256 (10)	−0.0063 (7)	−0.0028 (8)	0.0010 (8)
O6	0.0176 (9)	0.0234 (9)	0.0233 (9)	0.0033 (7)	−0.0022 (7)	−0.0020 (8)
O7	0.0267 (9)	0.0316 (10)	0.0253 (9)	0.0133 (8)	−0.0071 (7)	−0.0062 (8)
O8	0.0188 (9)	0.0258 (9)	0.0286 (9)	−0.0053 (7)	−0.0042 (7)	0.0044 (7)
O9	0.0210 (9)	0.0364 (10)	0.0197 (9)	−0.0048 (7)	0.0030 (7)	0.0009 (7)
O10	0.0202 (9)	0.0260 (9)	0.0249 (9)	0.0015 (7)	0.0027 (7)	−0.0049 (7)
Cl1	0.0288 (4)	0.0426 (4)	0.0458 (4)	−0.0069 (3)	−0.0045 (3)	0.0166 (3)
OW1	0.0298 (10)	0.0329 (11)	0.0349 (11)	0.0066 (8)	0.0000 (8)	0.0006 (9)

### Hexaquaaluminium sulfate chloride monohydrate (2) Geometric parameters (Å, º)

**Table:**

Al1—O3	1.8624 (17)	Al1—O2	1.8940 (17)
Al1—O5	1.8718 (18)	S1—O10	1.4670 (16)
Al1—O6	1.8752 (17)	S1—O9	1.4672 (16)
Al1—O1	1.8798 (17)	S1—O8	1.4753 (16)
Al1—O4	1.8855 (17)	S1—O7	1.4767 (16)
			
O3—Al1—O5	178.65 (8)	O5—Al1—O2	90.67 (8)
O3—Al1—O6	89.63 (8)	O6—Al1—O2	178.38 (8)
O5—Al1—O6	90.14 (8)	O1—Al1—O2	90.78 (8)
O3—Al1—O1	90.72 (8)	O4—Al1—O2	89.41 (7)
O5—Al1—O1	90.61 (8)	O10—S1—O9	110.75 (10)
O6—Al1—O1	90.61 (7)	O10—S1—O8	109.30 (10)
O3—Al1—O4	88.68 (8)	O9—S1—O8	109.04 (9)
O5—Al1—O4	89.99 (8)	O10—S1—O7	108.95 (9)
O6—Al1—O4	89.19 (7)	O9—S1—O7	109.71 (10)
O1—Al1—O4	179.37 (8)	O8—S1—O7	109.07 (10)
O3—Al1—O2	89.53 (8)		

### Hexaquaaluminium sulfate chloride monohydrate (2) Hydrogen-bond geometry (Å, º)

**Table:**

*D*—H···*A*	*D*—H	H···*A*	*D*···*A*	*D*—H···*A*
O1—H1···O9^i^	0.85 (1)	1.88 (1)	2.714 (2)	165 (3)
O1—H2···O8^ii^	0.85 (1)	1.85 (1)	2.690 (2)	170 (3)
O2—H3···O*W*1^iii^	0.85 (1)	1.85 (1)	2.692 (2)	178 (3)
O2—H4···O10	0.85 (1)	1.92 (1)	2.767 (2)	177 (3)
O3—H5···O7^iv^	0.85 (1)	1.78 (1)	2.629 (2)	174 (3)
O3—H6···O7	0.85 (1)	1.73 (1)	2.578 (2)	176 (3)
O4—H7···Cl1^v^	0.85 (1)	2.18 (1)	3.0311 (18)	177 (3)
O4—H8···O10^vi^	0.85 (1)	1.83 (1)	2.669 (2)	176 (3)
O5—H9···O*W*1	0.85 (1)	1.82 (1)	2.650 (2)	166 (3)
O5—H10···Cl1	0.85 (1)	2.17 (1)	3.0120 (18)	171 (3)
O6—H11···O8^vii^	0.85 (1)	1.83 (1)	2.672 (2)	172 (3)
O6—H12···O9^ii^	0.85 (1)	1.83 (1)	2.671 (2)	171 (3)
O*W*1—H13···Cl1^viii^	0.85 (1)	2.33 (2)	3.083 (2)	149 (3)
O*W*1—H14···Cl1^i^	0.84 (1)	2.68 (2)	3.390 (2)	143 (3)
O*W*1—H14···Cl1^iii^	0.84 (1)	2.74 (3)	3.280 (2)	123 (3)

Symmetry codes: (i) *x*, *y*, *z*+1; (ii) *x*+1, *y*, *z*+1; (iii) −*x*+2, −*y*+1, −*z*+2; (iv) *x*, −*y*+1/2, *z*+1/2; (v) −*x*+2, −*y*+1, −*z*+1; (vi) *x*+1, *y*, *z*; (vii) *x*+1, −*y*+1/2, *z*+1/2; (viii) −*x*+3, −*y*+1, −*z*+2.
